# A Retrospective Study to Evaluate the Perioperative Clinical Characteristics and Outcomes of Rhino-Orbital Cerebral Mucormycosis in COVID-19 Patients at a Tertiary Care Hospital in India

**DOI:** 10.7759/cureus.41613

**Published:** 2023-07-09

**Authors:** Rekha Kumari, Praveen Talawar, Debendra K Tripaty, Deepak Singla, Ashutosh Kaushal, Sameer Sharma, Manu Malhotra, Priyanka Boruah, Priyanka Sangadala, Karthikeyan S Kumar

**Affiliations:** 1 Anaesthesiology, All India Institute of Medical Sciences, Rishikesh, Rishikesh, IND; 2 Anaesthesiology, All India Institute of Medical Sciences, Raipur, Raipur, IND; 3 Anaesthesiology, All India Institute of Medical Sciences, Bhopal, Bhopal, IND; 4 Otorhinolaryngology & Head-Neck Surgery, All India Institute of Medical Sciences, Rishikesh, Rishikesh, IND; 5 Anaesthesiology, State Cancer Institute, Guwahati, Guwahati, IND

**Keywords:** rhino-orbito-cerebral mucormycosis, mortality, diabetes mellitus, perioperative outcome, covid-19

## Abstract

Background and aims

A descriptive analysis of patients who underwent surgical debridement for coronavirus disease 2019 (COVID-19) related mucormycosis was described, which aimed at the evaluation of perioperative clinical characteristics, perioperative complications, and outcomes.

Methods

We conducted a retrospective study on patients who underwent surgical intervention for mucormycosis during the COVID-19 pandemic at a tertiary care institute in India from March 1, 2021, to June 30, 2021. The medical records of 92 patients were reviewed and analyzed.

Results

There was a male predominance with a mean age of 50.86 years. The most common comorbidity was diabetes mellitus (DM) (98.9%). Intra-operative complications included hypotension, hyperglycemia, and hypokalemia. Most of the patients (88%) were extubated inside the operation theater, and 48% of patients had mortality. Serum ferritin levels, computed tomography severity score (CTSS), and D-dimers were significantly high in the patient who had mortality.

Conclusion

The perioperative mortality in patients with COVID-19 associated mucormycosis was very high. DM was the most common comorbidity followed by hypertension. Pre-operative elevated serum ferritin, D-dimer, and high CTSS were associated with higher mortality; hypokalemia, followed by hypocalcemia, was the most common perioperative and post-operative electrolyte imbalance. Thorough pre-operative optimization, multidisciplinary involvement, and perioperative care are of the utmost importance to decrease mortality and improve outcomes.

## Introduction

Coronavirus disease 2019 (COVID-19) was an emerging global public health event associated with a wide range of disease patterns, from mild to life-threatening pneumonia, associated with bacterial and fungal co-infections [1]. It was primarily managed with systemic corticosteroids and supportive care, mainly oxygen therapy. But unfortunately, the widespread use of corticosteroids leads to secondary bacterial or fungal infections and raised blood sugar levels [2].

Mucormycosis is the most common COVID-19 associated infection, which emerged as a matter of concern due to the high use of corticosteroids and uncontrolled diabetes mellitus (DM), especially in the Asian population [3,4]. The exposure to humidity and moisture during oxygen therapy while the patient is on mechanical ventilation creates a favorable environment for the mucoromycetes group of fungi causing mucormycosis. Therefore, during the second wave of the COVID-19 pandemic, there was a sudden surge in rhino-orbital-cerebral mucormycosis (ROCM).

Anesthesiologists were involved in the multidisciplinary management of ROCM as these patients require surgery under general anesthesia (GA) and require perioperative critical care [5]. The understanding of risk factors, management options, and factors affecting outcomes is vital in effective management. The present study was planned to retrospectively describe the characteristics, perioperative complications, and outcomes of patients undergoing surgical debridement.

## Materials and methods

This retrospective study was conducted in a tertiary care center in India after clearance from the Institutional Ethics Committee (IEC No: AIIMS/IEC/21/502, dated 09/10/2021) from March 1, 2021, to June 30, 2021, on COVID-19 patients who underwent surgical intervention under GA for mucormycosis during the COVID-19 pandemic. We have followed the STROBE (Strengthening the Reporting of Observational Studies in Epidemiology) guidelines to conduct this study. The hospital records of the study period were reviewed. Incomplete records and patients who were shifted outside of our makeshift hospital before completing the treatment were excluded from the analysis.

Medical records were screened for demographic profile (age, gender, American Society of Anesthesiologists physical status, any difficult airway by modified Mallampati score, comorbidities, and diagnosis and surgical procedure). Intra-operative course in terms of duration of surgery and anesthesia, type of induction agent, opioid, muscle relaxant, invasive monitoring requirement, blood loss, and whether patients were extubated in operation theatre or shifted to intensive care unit (ICU) were recorded. Post-operatively, patients’ records were recorded for the duration of hospital and ICU stay, post-operative oxygen requirement and mechanical ventilation, any electrolyte imbalances, documented organ failure and perioperative outcomes such as discharged, leave against medical advice (LAMA), or death.

The perioperative outcomes, i.e., mortality and survivors, were analyzed with respect to their pre-operative inflammatory markers (serum ferritin, D-dimer, and procalcitonin), CTSS, duration of hospital stay, duration of operation, and anesthesia. Additionally, we examined the relationship between the mean dose of liposomal amphotericin-B with mortality, post-operative complications, and the requirement for an ICU stay.

 Statistical analysis

Statistical data were entered into a Microsoft Excel sheet and analyzed using SPSS (Statistical Package for the Social Sciences) Version 16.0 (IBM Corp., Armonk, NY). Continuous parametric data were reported as mean and standard deviation, while continuous non-parametric data were reported as median and interquartile range. The association between pre-operative inflammatory markers and CTSS with perioperative outcomes (mortality) was compared by univariate and multivariate regression analysis. The association between the mean dose of amphotericin-B and perioperative outcomes was also analyzed in the same way.

## Results

**Figure 1:**
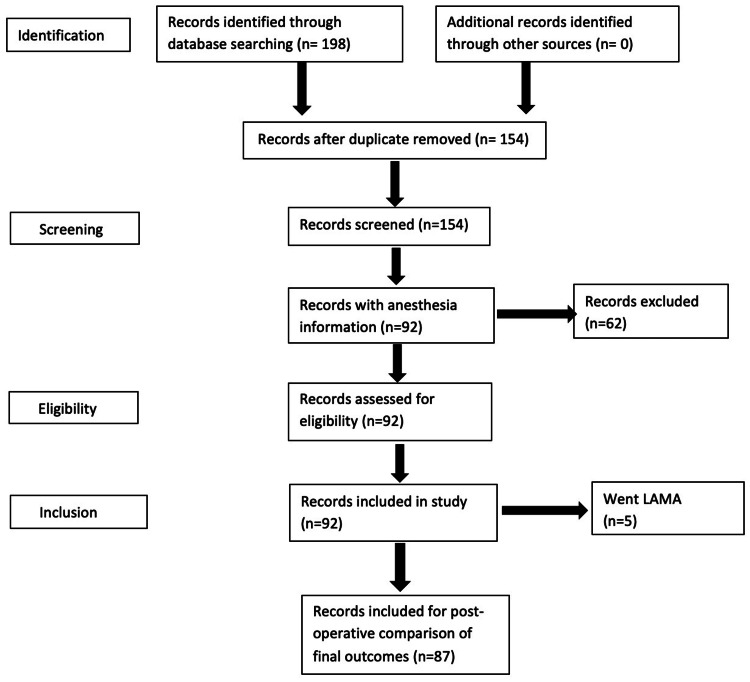
Methodology flow chart LAMA, leave against medical advice

A total of 198 records were identified, out of which 154 records were screened after removal of duplication, 92 medical records with anesthesia information were available as the rest of the patients were transferred to makeshift hospitals, and the records were not available; therefore, that was our sample size (n=92). Five patients took LAMA; therefore, post-operative records of 87 patients were reviewed for the final outcomes (Figure 1).

The pre-operative characteristics and demography are presented in Table 1.

**Table 1:** Demography and pre-operative characteristics FESS, functional endoscopic sinus surgery

Variable		Results
Number of patients:		92
Age in years (mean ± standard deviation)		50.86 ± 10.60
Sex (frequency and percentage)	Male	62 (67.4%)
	Female	30 (32.6%)
American Society of Anesthesiologist physical status (frequency and percentage)	II	35 (38%)
	III	52 (56.5%)
	IV	1 (1.08%)
Comorbidities (frequency and percentage)	Diabetes mellitus	91 (98.9%)
	Hypertension	26 (28.2%)
	Coronary artery disease	7 (7.6%)
	Hypothyroidism	6 (6.52%)
	Acute kidney injury	4 (4.34%)
	Chronic kidney disease	2 (2.17%)
	Asthma	1 (1.08%)
	Cerebrovascular accidents	1 (1.08%)
Type of surgery (frequency and percentage)	Open surgery (maxillectomy with orbital exenteration)	45 (45.9%)
	FESS	19 (20.65%)
	Combined (one side FESS + other side open surgery	28 (30.4%)

All patients received GA and were ventilated by controlled mechanical ventilation (volume-controlled mode) with 6-8 mL/kg of tidal volume. Intravenous induction was done with 2 mg/kg of propofol (94.5%) and 0.3 mg/kg of etomidate (5.43%) in cardiac patients; 0.1 mg/kg of vecuronium (89.13%), 1 mg/kg of succinylcholine (6.25%) and 0.5 mg/kg of atracurium in patients with deranged renal function (4.34%) was given as a muscle relaxant. The GA was maintained with sevoflurane and intermittent dose of muscle relaxant with oxygen and air with 2 liters/minute of fresh gas flow. All patients received crystalloids; 15.2% of patients also received colloids. Intra-operatively, 21.7% of patients developed complications such as hypotension (not responding to fluid therapy), which required vasopressors (norepinephrine 0.01-0.5 mcg/kg/minute) support (11.95%), hyperglycemia (5.43%), hypokalemia (3.21%), acidosis (1.08%), diabetic ketoacidosis (1.08%), and atrial fibrillation (1.08%). The intra-operative course is given in Table 2.

**Table 2:** Intraoperative course

Variables		Results
Duration of surgery (mean ± standard deviation) (minutes)		155 ± 49.56
Duration of anesthesia (mean ± standard deviation) (minutes)		170 ± 54.31
Type of intravenous induction agent (frequency and percentage)	Propofol	87 (94.5%)
	Etomidate	5 (5.43%)
Muscle relaxants (frequency and percentage)	Succinylcholine	6 (6.52%)
	Vecuronium	82 (89.13%)
	Atracurium	4 (4.34%)
Airway management (frequency and percentage)	Direct laryngoscopy	75 (81.52%)
	Video-laryngoscopy	16 (17.3%)
	Tracheostomy	1 (1.08%)
Invasive monitoring (frequency and percentage)	Central line	92 (100%)
	Arterial line	12 (13.04%)
Blood loss and transfusions	Blood loss in mL (mean ± standard deviation)	333.3 ± 171.32
	Blood transfusion (frequency and percentage)	23 (25%)
Extubation (frequency and percentage)	Yes	81 (88%)
	No	11 (11.9%)
Post-operative transfer (frequency and percentage)	Ward	81 (88%)
	Intensive care unit	11 (11.9%)

Post-operatively, the need for oxygen, re-intubation, electrolyte imbalances, and organ failure is given in Table 3.

**Table 3:** Post-operative course

Variables		Results
Hospital stay (mean ± standard deviation) (days)		23.32 ± 14.46
Intensive care unit stay (mean ± standard deviation) (days)		6.3 ± 6.75
Post-operative oxygen dependency in the immediate post-operative period (frequency and percentage)	Yes	26 (29.88%)
	No	66 (75.86%)
Re-intubation (frequency and percentage)		24 (27.5%)
Post-operative mechanical ventilation (frequency and percentage)	Yes	32 (36.7%)
	No	60 (68.9%)
Post-operative electrolyte imbalance (frequency and percentage)	Hypokalemia	65 (74.71%)
	Hypocalcaemia	59 (67.81%)
	Hyperkalemia	2 (2.29%)
Other organ failure (morbidity) (frequency and percentage)	Heart	4 (4.59%)
	Acute kidney injury	15 (17.2%)
	Chronic kidney disease (hemodialysis)	1 (1.14%)
	Brain (infarct)	1 (1.14%)
	Multiorgan failure and sepsis	16 (18.39%)
Outcome (frequency and percentage)	Discharge	45 (48.9%)
	Leave against medical advice	5 (5.43%)
	Death	42(48.27%)

A total of 48.27% of patients died while they were hospitalized. Univariate regression analysis for the association of inflammatory markers (serum ferritin, D-dimer, and serum procalcitonin) and CTSS with clinical outcome was statistically significant (Table 4).

**Table 4:** Univariate and multivariate logistic regression for mortality with pre-operative inflammatory markers and CTSS CTSS, computed tomography severity score

Variables		Results mean (standard deviation)		Univariate regression coefficient (95% confidence interval)	p-Value	Multivariate model statistics
		Mortality group	Survivor group			R square = 0.289; p-value = 0.880
Inflammatory markers	Serum ferritin (mcg/L)	611.25 (961.36)	130.75 (315.10)	1.001 (1.000–1.002)	0.010	
	D-dimer (mcg/ml)	2.21 (4.54)	0.59 (1.76)	1.190 (0.999–1.417)	0.051	
	Procalcitonin (ng/ml)	28.75 (70.26)	5.93 (12.03)	1.038 (0.985–1.094)	0.165	
CTSS		16.38 (4.24)	11.10 (3.21)	1.415 (1.044–1.917)	0.025	

The univariate regression analysis for the association of duration of hospital stay, duration of anesthesia, and surgery, with clinical outcomes was statistically significant (Table 5).

**Table 5:** Univariate and multivariate logistic regression for mortality with duration of hospital stay, surgery, and anesthesia

Patient parameter	Mean (standard deviation)		Univariate regression coefficient (95% confidence interval)	P-Value	Multivariate model statistics
	Mortality group	Survivor group			
Hospital stay duration	19.55 (12.64)	26.94 (16.29)	0.963 (0.932–0.996)	0.027	R square = 0.193; p-value = 0.376
Surgery duration	147.86 (47.01)	132.39 (48.30)	1.007 (0.998–1.016)	0.133	
Anesthesia duration	180.36 (51.91)	155.85 (56.62)	1.008 (1.000–1.017)	0.041	

The univariate regression analysis for the association between the cumulative amphotericin B dose with mortality, post-operative complications, and need for ICU stay was statistically significant (Table 6).

**Table 6:** Univariate and multivariate logistic regression for dose of Amphotericin B with patient outcomes

Patient outcome	Univariate regression coefficient (95% confidence interval)	p-Value	Multivariate model statistics
Mortality	700.37 (723.93–2124.67)	0.331	R square = 0.063; p-value = 0.036
Need for intensive care unit stay	1466.95 (35.996 - 2897.91)	0.045	
Post-operative complications	513.96 (462.31–1490.23)	0.298	

## Discussion

The anesthesia management of surgical debridement for mucormycosis is challenging due to the residual respiratory insufficiency, difficult airway, and the COVID-19 consequences in the post-operative phase. This retrospective study evaluated perioperative clinical characteristics, complications, and outcomes in patients who underwent surgical procedures for COVID-19 associated ROCM.

We found a male predominance of the disease, with a mean age of 50.86 years. In a study reported by Kayina et al. [6] in the Indian population, the mean age was 50.7 years, and 68.1% of the patients were males; the age and genders of our patients were consistent with our study. The male predominance may be explained by the likely protective effect of estrogens in females and the increased outdoor exposure in males, resulting in more fungus spores exposure [7].

DM is the most pre-dominant underlying medical comorbidity in patients with ROCM. In our study, 98.9% of patients had DM, consistent with the literature [8-10].

Managing mucormycosis involves control of hyperglycemia, surgical debridement, and medical management with antifungal agents. Amphotericin B is the antifungal drug of choice for mucormycosis. Our patients received liposomal amphotericin B and surgical debridement, and our results are consistent with the reported literature [7-9].

All the cases of ROCM were taken on an emergency basis; we had one operation theater exclusively for mucormycosis cases. The duration of surgery and anesthesia were in line with the reported literature [7,9-11]. The minor differences in time might be due to different surgical skills and logistic issues of particular institutes.

Amphotericin B-induced hypokalemia may enhance the effect of skeletal muscle relaxants; therefore, serum potassium levels should be monitored perioperatively [7,12]. Our patients received muscle relaxants according to their renal function. The incidence of difficult airways (36.9%) reported in the literature is in line with our study data [7,9,11]. In our study, 88% of patients were extubated after surgery in the operation theatre, and the remaining patients required prolonged mechanical ventilation in ICU due to post-COVID pulmonary sequelae, which was consistent with the findings reported by Karaaslan [10] and Sirohiya et al. [11] Initially, only 11.9% patients shifted to ICU post-operatively, and there were 27.5% patients who required re-intubation in the ward and were shifted to ICU later. The mean duration of hospital stay and ICU is consistent with the findings reported by Solanki et al. [9] and Karaaslan [10].

Sirohiya et al. [11] reported that the median time to hospital discharge was 53.5 days, which may be due to early presentation and the start of treatment at an early stage of the disease at the higher center where this study was conducted, which is reflected as lower mortality (12.28%) as compared to our research.

Amphotericin B causes nephrotoxicity and electrolyte imbalances; these were seen during their ICU stay in our study and were consistent with studies reported by Solanki et al. [9] and Meiring et al. [13].

COVID-19 associated ROCM is a fatal disease; a higher percentage of treatment failure and mortality were seen despite antifungal therapy and surgical debridement. Evidence suggests a 20-25% increase in 30-day mortality with an increased risk of post-operative pulmonary complications in patients undergoing surgery with perioperative COVID-19 infection [14]. In our study, mortality occurred in 42 (48.27%) patients during the hospital stay, which was consistent with the literature (Dave et al. [15] reported a mortality rate of 34%, Choksi et al. [16] reported 10-day mortality of 36% and 21-day mortality of 53%, and John et al. [17] reported a mortality rate of 49%). However, Sirohiya et al. [11] reported a mortality rate of 12.28%. The difference in mortality rates reported in the literature may be due to their different demographic profiles, comorbidities, immunocompromised state, some forms of mucormycosis (especially pulmonary and cerebral), severity of COVID-19, treatment received, the time of surgery, and overall care of ROCM in different institutes. Also, the COVID-19 and ROCM epidemics took us in a rapid way without giving us time to understand the disease’s nature (epidemiology) and plan appropriate management.

The inflammatory response, commonly known as “cytokine storm,” has been implicated in the progression and severity of infection in COVID-19 patients. The serum ferritin levels, a marker of immune dysregulation and an integral part of iron metabolism, were markedly elevated in COVID-19 patients [18-21], consistent with our study.

The D-dimer levels were found to be abnormally elevated in COVID-19 infection, and the same has been used as a prognostic marker in COVID-19. Zhang et al. [22] have shown that a four-fold increase at admission was found to predict in-hospital mortality in patients with COVID-19 infection. Similar findings were reported by Sirohiya et al. [11] and Hodges et al. [20]. In our study, D-dimer levels are consistent with the above literature. Pontia et al. [23] and Henry et al. [24] also reported that biomarkers raised in COVID mortality patients were C-reactive protein, D-dimer, and ferritin.

Early diagnosis is very important to isolate the patients, and computed tomography (CT) is thought to play an important role in COVID-19 diagnostic workup. Raoufi et al. [25] found that the mean (SD) of CTSS in a mortality group was higher and similar results ([26-28]) are in agreement with our study.

Patel et al. [29] conducted a multicentric observational study on 465 patients with COVID-19 associated ROCM; a higher survival was observed in patients who received combined medical and surgical treatment (p=0.001), which was statistically significant. Our results are consistent with the above study, concluding that amphotericin B decreases morbidity and mortality in COVID-19 associated ROCM.

The mean mortality time in the published literature ranges from 7 to 60 days [10,11,30]. In our study, the mean duration of hospital stay in a mortality group was 19.55 (12.64) days as compared to 26.94 (16.29) days in the non-mortality group, which is statistically significant (p=0.020), concluding that the severity of disease was more in the mortality group.

The prospective studies of anesthesia management and outcome could not be possible during the sudden surge of this type of cases of pandemic nature where the prognosis of COVID-19 itself was not known; hence, the retrospective study was undertaken. Our study has some limitations, including its retrospective character, the use of pre-existing data, and the fact that all patients came from a single center, which limit the generalizability of the findings; the mortality concerning a particular surgical procedure was not evaluated.

## Conclusions

The perioperative mortality in patients with COVID-19 associated ROCM was very high. This is likely the result of the various forms of the disease, the challenging diagnosis, especially for pulmonary mucormycosis, and the severity of COVID-19 infection. DM was the most common comorbidity, followed by hypertension, whereas hypokalemia, followed by hypocalcemia, was the most common perioperative electrolyte imbalance. The kidney was the most commonly involved organ with acute kidney injury presentation. Pre-operative elevated serum ferritin, D-dimer, and high CTSS were associated with higher mortality in ROCM patients. Therefore, this infection requires aggressive management with debridement and antifungal agents to salvage life. Anesthesia management requires a careful pre-operative evaluation and optimization of hyperglycemia, and a planned anesthetic administration coupled with strict hemodynamic monitoring and post-operative ICU care are of the utmost importance to decrease mortality and improve outcomes.
